# Worldwide population genetic structure of the oriental fruit moth (*Grapholita molesta*), a globally invasive pest

**DOI:** 10.1186/1472-6785-13-12

**Published:** 2013-03-25

**Authors:** Heather Kirk, Silvia Dorn, Dominique Mazzi

**Affiliations:** 1 ETH Zurich, Institute of Agricultural Sciences, Applied Entomology, Schmelzbergstrasse 9/LFO, Zurich 8092, Switzerland; 2 Current address: Institute of Systematic Botany, University of Zurich, Zollikerstrasse 107, Zurich 8008, Switzerland

**Keywords:** Microsatellites, Human-mediated dispersal, Isolation-by-distance, Invasive pest, Genetic structure, Population differentiation, Oriental fruit moth

## Abstract

**Background:**

Invasive pest species have large impacts on agricultural crop yields, and understanding their population dynamics is important for ensuring food security. The oriental fruit moth *Grapholita molesta* is a cosmopolitan pest of stone and pome fruit species including peach and apple, and historical records indicate that it has invaded North and South America, Europe, Australia and Africa from its putative native range in Asia over the past century.

**Results:**

We used 13 microsatellite loci, including nine newly developed markers, to characterize global population structure of *G. molesta*. Approximately 15 individuals from each of 26 globally distributed populations were genotyped. A weak but significant global pattern of isolation-by-distance was found, and *G. molesta* populations were geographically structured on a continental scale. Evidence does not support that *G. molesta* was introduced to North America from Japan as previously proposed. However, *G. molesta* was probably introduced from North America to The Azores, South Africa, and Brazil, and from East Asia to Australia. Shared ancestry was inferred between populations from Western Europe and from Brazil, although it remains unresolved whether an introduction occurred from Europe to Brazil, or vice versa. Both genetic diversity and levels of inbreeding were surprisingly high across the range of *G. molesta* and were not higher or lower overall in introduced areas compared to native areas. There is little evidence for multiple introductions to each continent (except in the case of South America), or for admixture between populations from different origins.

**Conclusions:**

Cross-continental introductions of *G. molesta* appear to be infrequent, which is surprising given its rapid worldwide expansion over the past century. We suggest that area-wide spread via transport of fruits and other plant materials is a major mechanism of ongoing invasion, and management efforts should therefore target local and regional farming communities and distribution networks.

## Background

Global interest in the ecology of agricultural systems has driven a substantial amount of fundamental research into plant-herbivore interactions, modes of plant (i.e. weed) and invertebrate dispersal and adaptation, and the role of multi-trophic interactions in community dynamics [1]. A growing need to understand the potential effects of climate change on cash crop production systems [2], combined with the necessity of increasing worldwide crop yields in response to projected global population growth [3], continue to motivate research into fundamental and applied questions in ecology and evolutionary biology.

The mechanisms that facilitate the establishment and spread of invasive pests are of particular interest to ecologists, evolutionary biologists and policy makers for a number of reasons; agricultural pests are responsible for substantial yield losses in agricultural systems, and there are considerable economic incentives to understand mechanisms of pest invasions. Also, because invasions often occur relatively rapidly, they provide useful case studies to understand the ecological factors (both biotic and abiotic) that mediate population dynamics and the establishment of range limits [4,5]. Finally, pest species often adapt to new selection pressures in their invaded ranges, and therefore represent contemporary examples of rapid evolutionary change [5,6].

The oriental fruit moth *Grapholita* (=*Cydia*) *molesta* Busck (Lepidoptera: Tortricidae) is one example of an insect pest species that continues to cause significant economic damage to crops on a global scale [7]. *Grapholita molesta* is a major pest of stone and pome fruit species mainly belonging to the Rosaceae family, including peach, apple, pear, nectarines, cherries, quince, and persimmons (Ebenaceae) [8,9]. Assumed to be native to China [7], *G. molesta* is now distributed throughout temperate regions of Asia, Europe, The Americas, Africa, and Australia.

The global demographic history of *G. molesta* has not been accurately traced, although historical records permit the reconstruction of a rough timeline of continental invasions. In Europe, *G. molesta* was first recorded in Slovenia in 1920 [10], in southeastern France and much of north-central Italy in the early 1920s [11], and has since dispersed throughout eastern, southern and western Europe, where stone fruit trees are grown. It is commonly assumed that the species was introduced to North America via a fruit shipment to Washington D.C. from Japan around 1913 [12], however this assumption is based on the anecdotal report of a single *G. molesta* specimen recovered from a shipment of Japanese pears [12]. After its introduction to Washington D.C., *G. molesta* is presumed to have dispersed to the neighboring states of Virginia and Maryland, and northward to Ontario, Canada. By the mid-1940s, it had spread from the mid-West to California, Washington state, and Oregon [13] and references therein]. In Australia, *G. molesta* was introduced around 1910, and has since spread to most of the stone and pome fruit growing regions on that continent [14] and references therein]. The species is assumed to have been present in southern Brazil since at least the 1940s [15]. In other parts of its range, introductions are thought to be more recent; *G. molesta* was first identified in New Zealand in 1976 [16], and in South Africa in 1980 [17].

A number of regional studies have yielded some information about local factors that contribute to the dispersal and population structuring of *G. molesta* over regional spatial scales. Population structure of *G. molesta* in a major fruit-growing region of Northern Italy was relatively low [18], and could be explained by both natural dispersal [19] and anthropogenic displacement of individuals between orchards [20]. Based on molecular genetic analysis of six populations in South Africa, it was also concluded that anthropogenic movement of fruit, bins, and nursery material likely drives range expansion at a regional level [21]. Another study showed that landscape features such as rivers might act as ecological barriers to regional dispersal [22]. In spite of substantial effort to understand the lifecycle of *G. molesta* and to develop strategies for its control, little is known about its ongoing global dispersal patterns.

In this study, we used a set of 13 microsatellite loci (including nine newly developed ones) to characterize global population structure of *G. molesta*, in order to retrace invasion routes, and to investigate global population dynamics. We hypothesized that i) patterns of population structure will reflect evidence of multiple introductions via human-mediated dispersal in the invaded range of the species, and ii) patterns of isolation-by-distance will be absent or weak, since international trade routes likely determine patterns of global population structure, and iii) genetic diversity will be reduced in introduced compared to native populations. We also tested whether populations are structured according to host species (predominantly peach and apple).

## Methods

### Insect sampling

**Figure 1:**
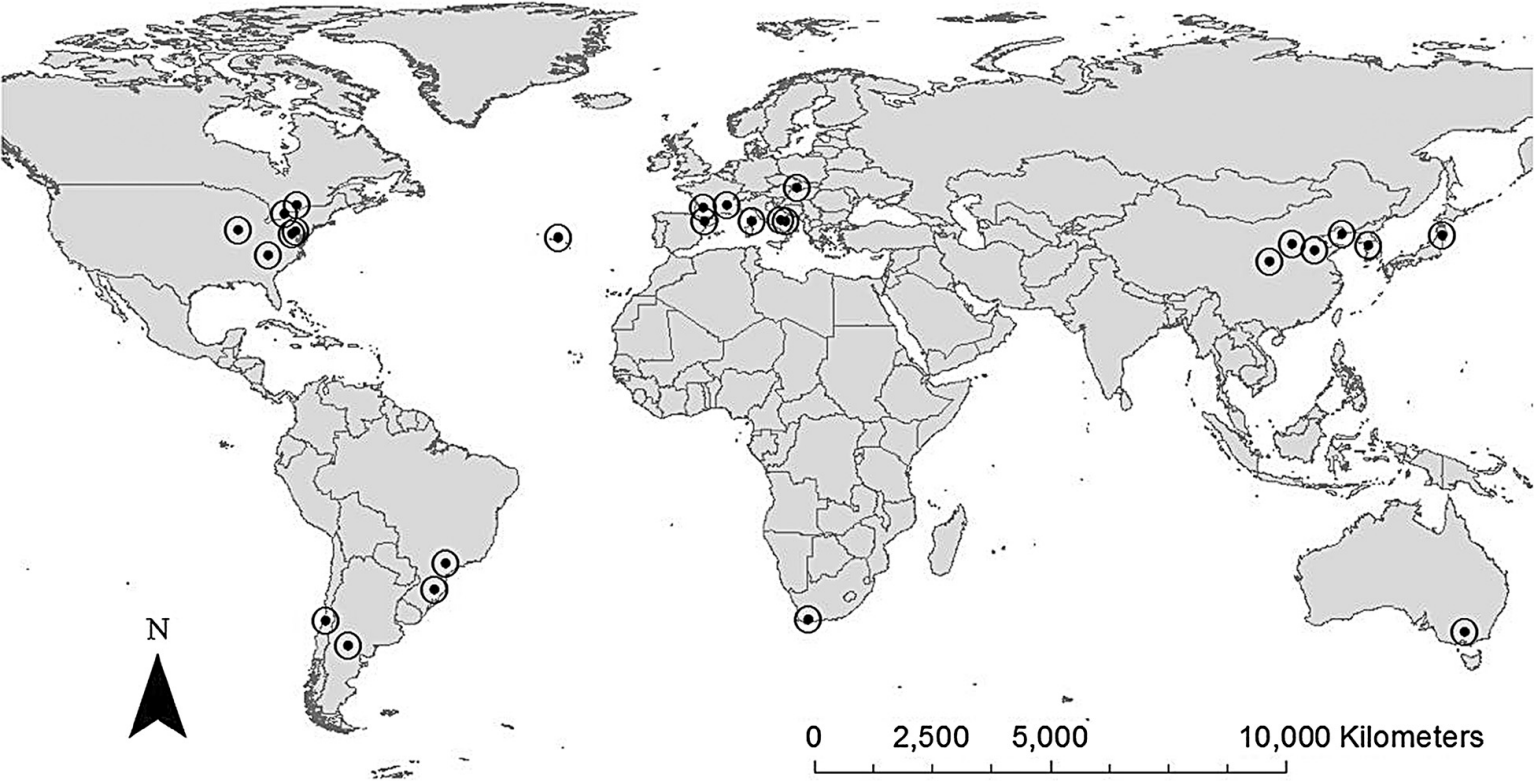
**Locations of 26 globally sampled *Grapholita molesta *populations.** Approximately 15 individuals were sampled from each population, and were genotyped at 13 microsatellite loci.

A total of 376 *G. molesta* larvae (sampled from fruits or shoots) or adult males (captured from pheromone traps) were collected from fruit trees at 26 worldwide locations between April and October 2011 (see Table 1; Figure 1). DNA has been stored, and voucher samples are deposited at the Entomological Collection of ETH Zurich. We sampled 15 individuals per site in most cases (Table 1). Upon sampling, geographical coordinates and host plant species were recorded. To maximize the genetic variability of larvae sampled within sites, we collected larvae from as many different trees as possible. Samples were stored in 70% ethanol.

**Table 1:** Sampling information

**Sampling location**	**Country**	**N**	**Collector/supplier**	**Host plant**	**Larvae/adults**	**Latitude**	**Longitude**
***North America***							
Champaign County, IL	USA	15	Richard Weinzierl	peach	larvae	40.098383	−88.213833
Mills River, NC	USA	15	James Welgenbach	peach	larvae	35.427210	−82.558880
Geneva, NY	USA	15	Arthur Agnello	apple	larvae	44.866360	−77.025430
Biglerville, PA	USA	15	Greg Krawczyk	peach	larvae	39.931694	−77.254531
Kearneysville, WV	USA	12	Brent Short	apple	adults	39.358317	−77.893889
Vineland Station, ON	Canada	15	Leo Van Driel	peach	larvae	43.170808	−79.394003
***Asia***							
Feicheng, Shandong Province	China	15	Maohua Chen	pear	adults	36.233333	116.766667
Yangling, Shaanxi Province	China	15	Maohua Chen	pear	adults	34.266667	108.066667
Taigu, Shanxi Province	China	15	Maohua Chen	pear	larvae	37.433333	112.533333
Pulandian, Dalian Liaoning Province	China	15	Jiang Xiaolong	peach	larvae	39.394378	121.963222
Kitakami, Iwate	Japan	15	Hiroshi Hada	apple	adults	39.286764	141.113192
Gyeonggi-do	South Korea	15	Minyoung Kim	peach	adults	37.266611	126.976664
***Europe***							
Campomarino (Campobasso)	Italy	15	Pasquale Trematerra	peach	larvae	41.957056	15.034703
Vieste	Italy	12	Dominique Mazzi	peach	larvae	41.881803	16.173369
St. Marcel-lès-Valence	France	15	Sylvaine Simon	peach	adults	44.971933	4.956883
Lafitte-sur-Lot	France	15	Dolors Bosch	apple	larvae	44.362833	0.463167
Vràble	Slovakia	15	Peter Tòth	peach	larvae	48.243731	18.308203
Prvacina	Slovenia	15	Mojca Rot	peach	both	41.769782	9.596859
La Portella, Lleida	Spain	15	Dolors Bosch	peach	larvae	41.752917	0.635000
Terceira Island	Azores	15	David João Horta Lopes, Reinaldo Macedo Soares Pimentel	peach	larvae	38.721642	−27.220578
***Africa***							
Swellendam	South Africa	5	Monique Rentel	Sample A peaches	adults	−34.016667	20.433333
		3	Monique Rentel	Sample B persimmon	adults	−34.016667	20.433333
***Australia***							
Greater Shepparton	Australia	15	Alex Il’ichev	peach	larvae	−36.500000	145.350000
***South America***							
Vacaria, Rio Grande do Sul	Brazil	15	Priscila Strapasson	apple	larvae	−28.510153	−50.930364
Campos de Holambra	Brazil	15	Marcos Botton	peach	larvae	−23.388611	−48.722778
Requínoa	Chile	13	Estaban Basoalto Venegas	peach	adults	−34.323817	−71.53395
Cervantes	Argentina	15	Esteban Tudela	peach	larvae	−39.063139	−67.360747

### DNA Extraction and Simple Sequence Repeat (SSR) genotyping

Genomic DNA was extracted from 8–10 mg larvae or adult male moths (heads and thoraxes) (see Table 1) using the Nucleospin Genomic DNA from Tissue Kit (Macherey-Nagel AG, Oensingen, Switzerland). Extraction was performed according to the bench protocol for animal tissues. In the final step, DNA was eluted in 200 μL TE buffer and stored at −20°C.

A total of 13 SSR loci were used for genotyping. Primer sequences and reaction conditions for the amplification of four of these loci were previously described [18], and are given in Table 2. A new set of nine SSR markers was developed by ecogenics GmbH (Zurich-Schlieren, Switzerland) using the high-throughput genomic sequencing approach described by [23]. 1.25 μL of genomic DNA were analyzed on a Roche 454 GS-FLX platform (Roche, Basel, Switzerland) using a 1/16^th^ run and the GS-FLX titanium reagents. Of these, 2 183 contained a microsatellite insert with a tetra- or a trinucleotide of at least six repeat units or a dinucleotide of at least 10 repeat units. Suitable primer design was possible in 564 reads, of which 36 were tested for polymorphism in fifteen unrelated individuals derived from geographically disparate populations. Of these, 27 were eliminated because they showed failures or high null allele frequencies, were not polymorphic, or had complex peak patterns that could not be reliably interpreted. Characteristics of the newly developed microsatellite DNA markers are given in Table 2.

**Table 2:** **Characteristics of 13 microsatellite loci in *Grapholita molesta***

**Locus**	**GenBank number**	**Primer sequences 5 **^**′**^**- 3 **^**′**^	**Size range**	**Number of alleles**	**Null allele frequency**^**1**^	***T***_**a **_**(°C)**
GM02*	HM177460	F: CTCAGACCTGAGGGAACGAC	75-117	19	0.17	56
		R: CAACACACAGTAAGTTGAGTTTTGTC				
GM05*	HM177463	F: CAAGCAGTAATCGCAAACATC	150-222	28	0.25	56
		R: TGAGGACCAAGATGGTAGACAC				
GM07*	HM177465	F: GCAGGAAGCGATACTGCAAC	82-94	7	0.07	50
		R: GAAGCATCGAACCTTGTCG				
GM10*	HM177468	F: GTAGCGTTGACAGGCGTTG	158-202	24	0.13	50
		R: TGCGTTTACTTAGAGTATCTGTGC				
*GM11*	KC573059	F: GATCGCCGAATCAACTTCCC	213-295	19	0.24	56
		R: CACAATACTAAGAGTAGGATCTAGTGC				
*GM12*	KC573060	F: GACCTAGTTAGAGTCGCGGG	212-240	8	0.27	56
		R: CAAGGAGTTGGGTTGGTTGG				
*GM13*	KC573061	F: ACACTTCTTCATTTTATCCGTCTC	113-145	9	0.21	56
		R: TTATACGAAATAGACATGTGTGGG				
*GM14*	KC573062	F: GCAGTGGACGTCTTAACGC	131-163	9	0.18	56
		R: TGTAGGTACTTGACTTCCAAATGC				
*GM15*	KC573063	F: CCTACCTCTACTAGTCACACCC	140-166	16	0.05	56
		R: CGCGTGGAGTAACCTTGAAC				
*GM17*	KC573064	F: CGACATGTGGAACTGTCTAAC	230-292	18	0.33	56
		R: TCCTCTGAGAAATCGCACCG				
*GM18*	KC573065	F: GGAGTTCATCAAGTCTCAGCG	108-140	8	0.25	56
		R: ACTTTGCTCCCTTCGTATAGC				
*GM20*	KC573066	F: GTACCTACAGATCTCACAAGTATTAAC	196-248	23	0.27	56
		R: GAAGAACCATGTACGGCAGG				
*GM21*	KC573067	F: AAAGTGATGTCGTCCGTGAG	193-255	27	0.33	56
		R: TGCATAACGTGTGTAAGAAAGTG				

^1^Estimated in Geneland.

F: forward primer, R: reverse primer, bp: base pairs, *T*_a_: annealing temperature.

*from [18].

All polymerase chain reactions (PCRs) were performed as singleplex reactions. Reactions were performed in a Labcycler thermocycler (Sensoquest GmbH, Göttingen, Germany) in a total reaction volume of 10 μL. The forward primers of the loci *GM02*, *GM05*, *GM07*, and *GM10* were fluorescently labeled with joe, fam, fam, and hex respectively. Reactions for these four loci were carried out in a mix of 5.4 μL ddH_2_O, 1.0 μL PCR buffer (Qiagen, Hombrechtikon, Switzerland), 2.0 μL dNTPs (2.5 mM, Qiagen), 0.5 μL of each forward and reverse primer (2 μM), 0.1 μL HotstarTaq (5U, Qiagen) and 0.5 μL DNA (10–35 ng⁄ μL). Reactions for the remaining nine newly developed loci were carried out in a mix of 5.7 μL ddH_2_O, 1.0 μL PCR buffer (Qiagen), 1.0 μL dNTPs (2.5 mM, Qiagen), 0.2 μL of each forward primer (2 μM), 0.5 μL of each reverse primer (2 μM), 0.5 μL M13 primer (2 μM, fluorescently labeled with fam, Microsynth, Balgach, Switzerland), 0.1 μL HotstarTaq (5U, Qiagen) and 0.5 μL DNA (10–35 ng⁄ μL). Locus-specific PCR conditions are reported in Additional file 1: Appendix 1.

Genotyping was carried out by denaturing 1 μL PCR product in 9 μL formamide (deionized, 99.5% minimum, Sigma-Aldrich, Buchs, Switzerland) with 0.09 μL GeneScan 500 LIZ size standard (Applied Biosystems, Foster City, CA, USA), and running the products on a laser detection system (3730xl DNA Analyzer, Applied Biosystems). DNA sizing and allele definitions were performed using GeneMapper 4.0 software (Applied Biosystems). SSR data are archived at the Dryad repository (http://dx.doi.org/10.5061/dryad.h658g).

#### Statistical Analyses

We identified departures from Hardy–Weinberg equilibrium (HWE) using GenAlEx v. 6.41 [24]. We calculated null allele frequencies using the R package Geneland 3.1.4 [25,26]. We tested for linkage disequilibrium between all pairs of loci using Arlequin 3.01 [27], according to [28].

### Population genetic structure

Genetic and geographic distances were calculated in GenAlEx using a Euclidean distance metric according to [29], and missing data were interpolated by the software. A test of isolation-by-distance was carried out by applying a Mantel’s test to matrices of genetic and geographic distances in GenAlEx using 999 permutations.

To better understand the partitioning of regional population genetic structure, we carried out an AMOVA in GenAlEx, using 999 permutations, and also calculated global (mean) *F*_ST_ value between populations [30]. We defined populations according to the sample sites listed in Table 1, and we defined eight separate regions: North America, Europe (excluding The Azores), Asia (excluding Japan), South America, Australia, South Africa, Japan, and the Azores. Japan and the Azores were included as separate regions because we hypothesized that island populations may be significantly differentiated from mainland populations. In order to determine whether host plant species accounted for a portion of the genetic variation, we carried out an additional AMOVA for which we defined “region” as host plant (apple or peach). We removed three samples that were collected from persimmon (South Africa), and 45 samples that were collected from pear. The latter samples were collected from three populations in China, and are therefore not representative of a broad geographic sampling range. The final analysis contained 328 samples, 256 of which were collected from peach, and 72 of which were collected from apple.

**Figure 2:**
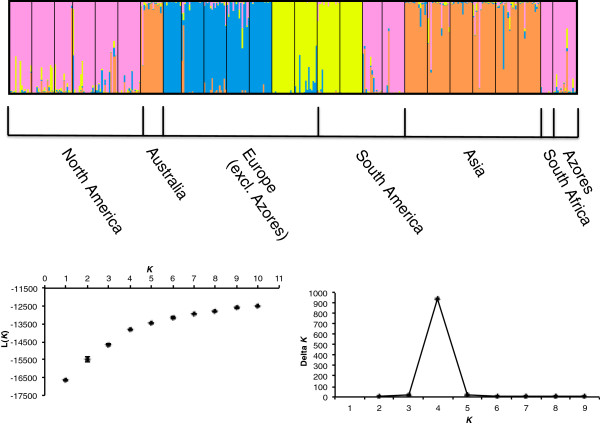
**Bayesian clustering according to the software program Structure; results for *K*=4.** Each individual is represented by a line, which is partitioned into *K* colored segments according to the individuals’ estimated membership fractions in each of the *K* clusters. Graphs show mean L(*K*) (±SD) over 5 runs for each value of *K* between 1 and 10, and Δ*K* calculated according to [34].

A Bayesian model-based analysis was performed to infer population structure using Structure version 2.3 [31,32]. Although Structure assumes HWE and no linkage disequilibrium between loci, the software is robust to deviations from these assumptions, particularly if no spurious populations are observed across multiple runs [33]. Data were analyzed using an admixture ancestry model with correlated allele frequencies, to estimate the posterior probabilities L(*K*) of *K* groups and the individual percentages of membership assigned to them according to their molecular multilocus profiles [32]. We examined the probabilities for a range of *K* (*K* = 1–10), using a burn-in period and a run length of the Markov Chain Monte Carlo (MCMC) of 30 000 and 100 000 iterations, respectively. Based on a trial run, we found that incorporating higher values of *K*, and longer burn-ins or MCMC did not appreciably change the results. Five runs were carried out for each value of *K*. We calculated Δ*K* according to [34]. Figure 2 was drawn using the program Distruct[35].

We also carried out an independent analysis of spatial structure using the R package Geneland 3.1.4 [25,26], which has been explicitly tested for robustness to the presence of null alleles. Like Structure, the software uses a MCMC strategy [36] to determine the most likely number of populations (*K*), and assigns individuals to the most appropriate population basing on individual multilocus genotypes. We carried out ten independent MCMC simulations (100 000 iterations, thinning of 100 iterations) using the non-spatial approach, during which we allowed *K* to vary between 1 and 20. Afterwards, the model with the highest mean logarithm of posterior probability was post-processed. Geneland was used to estimate inbreeding coefficients (*F*_IS_) of inferred clusters.

We also attempted to estimate the magnitude and direction of historical gene flow between our 26 sample populations using a Bayesian coalescent approach implemented in the software program Migrate-*N*[37]. However the lower 0.025 posterior distribution value of the pairwise migration estimates (M) never differed from 0. In other words, migration rates between populations never differed significantly from zero, and the direction of gene flow between sampling locations could not be inferred using this approach (results not shown).

### Genetic diversity

We calculated expected heterozygosity (*H*_E_), observed heterozygosity (*H*_O_), and allelic richness (*N*_A_) using GenAlEx. GenAlEx was used to identify private alleles from each continent, and from Japan and the Azores.

## Results

All of the nine newly developed markers (Table 2) proved to be polymorphic and informative. Among the 13 microsatellite loci used for genotyping, the number of alleles per locus ranged from 7 to 28 (Table 2). There were significant deviations from HWE at multiple loci from all sampling locations (data not shown), and in all cases these were the result of heterozygote deficiencies (Table 3). Null allele frequencies varied from 0.05 to 0.33 among the 13 microsatellite loci (Table 2), which is typical for lepidopteran DNA loci [38], and lower than or comparable to other studies that have used microsatellite markers to determine the genetic structure of populations [39,40]. Significant linkage disequilibrium was present in 15 out of 78 pairs of loci overall, but was unlikely due to physical linkage; patterns of significant allelic associations were not consistently restricted to certain pairs of loci within the 26 populations (data not shown).

**Table 3:** **Number of sampled individuals (*N*), inbreeding coefficient** (*F*_***IS***_), **expected heterozygosity (*H***_**E**_**), observed heterozygosity (*H***_**O**_**), and allelic diversity (*N***_**A**_**; corrected for sample size) of *Grapholita molesta *individuals from 26 sampling sites**

**Geneland cluster**	**Sampling location**	***N***	***F***_**IS**_	***H***_**E**_	***H***_**O**_	***N***_**A**_
1	All North American populations	87	0.493	0.618	0.316	7.46
2	European populations excluding Lafitte-sur-Lot (France), La Portella, (Spain), and The Azores	72	0.514	0.622	0.306	7.00
3	The Azores	16	0.338	0.551	0.381	4.69
4	La Portella, (Spain)	15	0.433	0.331	0.280	2.23
5	Lafitte-sur-Lot France Vacaria, (Brazil)	30	0.417	0.537	0.258	4.00
6	Campos de Holambra, (Brazil)	15	0.208	0.232	0.191	2.00
7	Argentina and Chile	28	0.545	0.524	0.246	4.54
8	Feicheng and Taigu, (China)	30	0.412	0.716	0.432	8.77
9	Yangling, (China)	15	0.373	0.532	0350	4.54
10	Pulandian, (China)	15	0.426	0.696	0.420	6.85
11	South Korea	15	0.384	0.699	0.453	6.92
12	Japan	15	0.455	0.616	0.357	4.85
13	South Africa	8	0.354	0.192	0.137	1.92
14	Australia	15	0.519	0.684	0.347	5.31

### Population genetic structure

Global *F*_ST_ across the data set was 0.219. AMOVA results showed that variation within populations, between populations, and among regions accounted for 72%, 18%, and 10% of the total variation, respectively (*P* < 0.001). Incorporating host species (peach or apple) rather than geographic region as the “regional” factor revealed that host species did not account for any of the variation in the data set, while variation within and between populations accounted for 70% and 30% of the variation, respectively (*P* < 0.001). A significant pattern of isolation-by-distance was detected (*P* < 0.001), although this pattern explained a low proportion of the variation in the data set (*R*^2^ = 0.111).

Bayesian analysis of population genetic structure conducted with the program Structure yielded a modal value of Δ*K* at *K* = 4 (Figure 2), and different runs at *K* = 4 produced consistent clustering solutions (Figure 2). Populations from the Azores, South Africa, Argentina, and Chile clustered with populations from North America. Australian and Japanese populations clustered with populations from mainland Asia. Most European populations clustered together, except for single populations from France (Lafitte-sur-Lot) and Spain, which clustered with populations from Brazil. There was little evidence for substantial admixture between populations that clustered separately (Figure 2).

Based on analysis in Geneland, the most likely number of inferred populations was *K* = 14 consistently across all ten independent runs (Table 3). Although the results from Geneland indicated a greater degree of population structure compared to the Structure results, there were no qualitative disagreements between the two analyses. In other words, populations that clustered together based on the Geneland analysis (Table 3) always clustered together in the Structure analysis (Figure 2). Pairwise *F*_ST_ values between clusters derived from the Geneland analysis are reported in Additional file 2: Appendix 2.

In order to further investigate the relationship between the two Brazilian and the two European populations that clustered together based on the Structure analysis, we used the private allele function in GenAlEx to check whether the sets of alleles from the two Brazilian populations were a subset of the alleles found in France (Lafitte-sur-Lot) and Spain, or vice versa, which would allow us to infer the direction of gene flow between continents. We found a similar number of private alleles among the Brazilian versus the French/Spanish populations; compared to Brazilian populations, there were 11 alleles that were specific to France (Lafitte-sur-Lot)/Spain, with allele frequencies that varied between 0.033, and 0.310. Similarly, there were 13 alleles that were specific to Brazil compared to France (Lafitte-sur-Lot)/Spain, with allele frequencies that varied between 0.017 and 0.433. The direction of gene flow between Brazil and Europe could therefore not be unambiguously traced using molecular genetic data.

### Genetic diversity and inbreeding

Measures of genetic diversity were high in native as well as in many introduced populations (Table 3). Measures of genetic diversity were substantially lower in one population from Brazil (Geneland Cluster 6), one population from Spain (Geneland Cluster 4), and the South African population (Geneland Cluster 13).

The number of private alleles was more than six times greater among Asian populations compared to European and North American populations (Table 4), even though fewer individuals and populations were sampled from Asia compared to either of the latter two continents. *F*_IS_ estimates corrected for null alleles (computed using Geneland), were high across the range of sampled populations, and did not differ appreciably among populations from the introduced versus invaded ranges (Table 3).

**Table 4:** Private alleles from each geographic region

	**Pop *N***^**1**^	***N***^**2**^	**Private alleles**
North America	6	87	7
Europe (excluding Azores)	7	102	10
Asia (exluding Japan)	5	75	62
Australia	1	15	2
South Africa	1	8	0
South America	4	58	8
Azores	1	16	1
Japan	1	15	1

^1^Number of populations sampled.

^2^Number of individuals sampled.

## Discussion

*Grapholita molesta* populations are structured on a global scale, and geographic structure generally reflects continental divisions; these data, combined with knowledge gleaned from historical records, provide important information regarding dispersal and population genetic structure of this invasive species throughout its range.

### Population genetic structure and migration

Populations were highly differentiated, with a global *F*_ST_ value of 0.219. In comparison, population genetic structure of the codling moth (*Cydia pomonella*), which belongs to the same tribe (Grapholitini) as *G. molesta*[41] and is also a major pest of fruit crops, varies from absent/low (France [42]; Chile [43]) to moderate (Switzerland [44]) to high (South Africa [45]; Italy [46]) among different geographic areas. However, the present study was carried out at a global scale, while individual studies of *C. pomonella* were carried out at scales varying from a few to several hundred kilometers, impairing comparisons between the two species. Nonetheless, low levels of population structure among *C. pomonella* populations from some regions relative to *G. molesta* could be the combined result of higher dispersal abilities of the former species compared to the latter [19,47-49] and the frequent use of pesticides against *C. pomonella* in some countries, which can stimulate flight in this species (reviewed by [44]).

In the present study, clustering outcomes from two distinct Bayesian approaches were largely congruent, except that the analysis from Geneland suggested greater population substructure compared to the analysis from Structure. Evidence indicates that *G. molesta* was introduced to Australia from mainland Asia. The data also support a North American source of *G. molesta* in Argentina and Chile, The Azores and South Africa, since historical records indicate that *G. molesta* was present in North America from the beginning of the 20^th^ century, but arrived much later in the latter three regions. However, a previous study [21] suggested that South African populations were unlikely to be derived from Canadian populations, since the genetic distance estimates from these two regions were relatively high.

Unexpectedly, Brazilian populations clustered together with two European populations (Structure), and shared ancestry between Western European and Brazilian populations was also supported by results from Geneland. The two European populations that shared ancestry with populations from Brazil showed little evidence of admixture with other European populations. It is possible that these two populations from Europe were derived from the same source as other European populations, but were isolated from other populations on the same continent, and therefore became differentiated over time. If this is the case, Brazilian populations are likely derived from European origins. However, it is also possible that *G. molesta* was recently re-introduced to Europe from the South American part of its range. Chile is the primary source of off-season peach and nectarine imports to Europe, and Brazil exports nearly 90% of its apple crop to Europe [50,51]. In contrast, stone and pome fruit exports from Europe to South America are negligible [50,51]. This latter interpretation of our data is in line with recent findings [42] of *C. pomonella* individuals with putative South American origin in France, suggesting that South America may be a common source of introduction for important stone and pome fruit pests to Western Europe. Moreover, other invertebrate pests of distantly related plant host species have recently been introduced from South America to Europe, including the pine-infesting woodwasp *Sirex noctilio*, which was introduced from Chile to Switzerland [52]. Although most invasive species in Europe are assumed to originate from North America or Asia, there is growing evidence that a number of invasive invertebrates in Europe come from South America (e.g. [42], [52], this manuscript).

Although it has been suggested that *G. molesta* was introduced to North America from Japan [7,12,13,53], we did not find evidence to support this hypothesis. The single Japanese population incorporated in this study clustered with other Asian populations, and did not show evidence of shared ancestry with North American populations. However, it is possible that limited sampling of populations in Japan prevented us from capturing a likely source population from that region, or that a historical bottleneck followed by high rates of mutation since the North American introduction have generated divergence levels that prevent the detection of shared ancestry. Our data did not provide an alternative hypothesis regarding the source of North American *G. molesta* populations.

We found an overall (albeit weak) pattern of isolation-by-distance, suggesting that regional introductions occur in a relatively stepwise manner. Also, we found little evidence for substantial admixture between populations sampled from different continents, which, combined with the clustering outcomes, implies that multiple cross-continental introductions are unlikely. We note however, that sampling from Australia and Africa was limited to one population each, and we cannot make definitive continent-wide conclusions for these regions based on these sample sizes.

It has earlier been suggested that *G. molesta* disperses primarily through the movement of fruit, bins and plant material between orchards [18,21]. The natural dispersal ability of both males and females is limited, even though certain environmental factors can increase flight capacity [54]. Flights between non-contiguous orchards are possible, however they are generally short [19,47], and human-mediated dispersal is implicated as the main mode of dispersal [20]. The poor natural dispersal ability of *G. molesta* likely accounts for the high levels of inbreeding observed in our study, since close relatives are probably geographically constrained to the same or neighboring orchards. Combined, these findings suggest that improved management of this invasive pest at regional and community levels (e.g. by area-wide pest management) and at the national level (e.g. by quarantine regulations) may be efficient strategies to limit its ongoing spread.

We did not find any evidence that *G. molesta* populations are structured according to host plant species. Although *G. molesta* has been shown to perform better on peach, its primary host, compared to apple [55], serial generations of a population sometimes infest different hosts as the growing season progresses [56]. Such seasonal host shifts may preclude adaptation of populations to any specific host, particularly if several suitable hosts are available within close geographic proximity.

### Genetic diversity and inbreeding

Estimates of genetic diversity among different geographic regions were high overall. A six times higher number of private alleles among Asian populations compared to populations from other continents provides, for the first time, convincing evidence that Asia is indeed the native range of this species, as is frequently suggested in the literature. We found that genetic diversity levels are surprisingly high across most of the invaded range, except for a few populations (Brazil, The Azores, and South Africa). It is therefore unlikely that founder effects impair the adaptive potential of *G. molesta* in most non-native areas, as is suggested to occur in other invasive species. Levels of genetic diversity were reported from the non-native range of 14 species of invasive insects [6], and of these only four species (*Ceratitis rosa*, *Drosophila pseudoobscura*, *Polistes dominulus*, and *Rhagoletis completa*) exhibited *H*_E_ values above 0.3. In our study, *H*_E_ exceeded 0.5 in most populations from both the native and invaded range. High levels of genetic diversity observed in this study could be a result of high microsatellite mutation rates in lepidopterans [57], and/or may result from the introduction of many individuals at each founding event.

High levels of genetic diversity may account for high levels of heritable phenotypic variation within *G. molesta* populations, and may contribute to the ability of *G. molesta* to adapt to pest management regimes. For example, there is considerable genetic variation within *G. molesta* populations with regard to olfactory response of female moths to their host plants [58]. Similarly, intra- and inter-population variation in other traits may enable adaptation to new environments and/or pest control regimes, although heritable variation in such traits has not yet been quantified. Nonetheless, a number of authors have demonstrated that *G. molesta* has developed insecticide resistance in at least some parts of its invaded range [59,60].

## Conclusions

Unlike many other invasive pests (reviewed by [61]), which have high dispersal abilities and/or are otherwise predisposed to multiple introductions, we found little evidence for frequent introductions between different continents in the case of *G. molesta*, with the possible exception of a reintroduction to Europe from South America, or two independent introductions to South America. This is likely a result of the poor natural dispersal abilities of *G. molesta*, combined with stringent international trade regulations that aim to prevent its movement across international borders. Although bottlenecks have occurred in a subset of introduced populations, we argue that, overall, reductions in genetic diversity will not prevent *G. molesta* from adapting to new control measures or changes in climatic conditions, and we therefore suggest that improved community-based control measures, in addition to more stringent national and intracontinental regulations regarding the regional transport of plant material, will be most effective for containing its ongoing spread.

## Competing interests

The authors declare that they have no competing interests.

## Authors’ contributions

SD and DM conceived of and designed the study. HK carried out the molecular genetic work, analyzed the data, and drafted the manuscript. All authors read and approved the final manuscript.

## Supplementary Material

Additional file 1 **Appendix 1.** Locus specific PCR conditions. Description: Detailed description of PCR conditions. Click here for file

Additional file 2 **Appendix 2.** Pairwise FST values. Description: Pairwise FST values from 14 clusters derived from analysis in GENELAND. Click here for file
