# Supplementary material for: Worldwide population genetic structure of the oriental fruit moth (Grapholita molesta), a globally invasive pest
Source: BMC Ecol. 2013 Mar 25;13:12. doi: 10.1186/1472-6785-13-12 (PMC3637152; doi:10.1186/1472-6785-13-12)
Supplement: Additional file 1 — Appendix 1. Locus specific PCR conditions. Description: Detailed description of PCR conditions. [file 1472-6785-13-12-S1.docx]

**Appendix 1.** Reaction Conditions for PCR amplifications

Loci *GM02*, *GM05*, *GM07*, *GM10*

The thermocycle consisted of an initial denaturation of 15 min at 95 °C, followed by 30 cycles consisting of 30 s at 95 °C, 45 s at annealing temperature (50 or 56 °C depending on the marker; see Table 1) and 45 s at 72 °C. The final extension was carried out for 10 min at 72 °C.

Loci *GM11*, *GM12*, *GM13*, *GM14*, *GM15*, *GM17*, *GM18*, *GM20*, *GM21*

The thermocycle consisted of an initial denaturation of 15 min at 95 °C, followed by 30 cycles consisting of 30 s at 95 °C, 45 s at annealing temperature (50 or 56 °C depending on the marker; see Table 1) and 45 s at 72 °C. Subsequently eight cycles were carried out that consisted of 30 s at 95 °C, 45 s at 53 °C (the annealing temperature of the M13 primer) and 45 s at 72 °C. A final extension of 30 min was carried out at 72 °C.
