# Supplementary material for: Worldwide population genetic structure of the oriental fruit moth (Grapholita molesta), a globally invasive pest
Source: BMC Ecol. 2013 Mar 25;13:12. doi: 10.1186/1472-6785-13-12 (PMC3637152; doi:10.1186/1472-6785-13-12)
Supplement: Additional file 2 — Appendix 2. Pairwise FST values. Description: Pairwise FST values from 14 clusters derived from analysis in GENELAND. [file 1472-6785-13-12-S2.docx]

| **Geneland Cluster** | | **1** | **2** | **3** | **4** | **5** | **6** | **7** | **8** | **9** | **10** | **11** | **12** | **13** | **14** |
| --- | --- | --- | --- | --- | --- | --- | --- | --- | --- | --- | --- | --- | --- | --- | --- |
| **1** | All North American populations | 0.000 |  |  |  |  |  |  |  |  |  |  |  |  |  |
| **2** | European populations excluding Lafitte-sur-Lot, France, La Portella, Spain, and The Azores | 0.156 | 0.000 |  |  |  |  |  |  |  |  |  |  |  |  |
| **3** | The Azores | 0.103 | 0.187 | 0.000 |  |  |  |  |  |  |  |  |  |  |  |
| **4** | La Portella, Spain | 0.243 | 0.319 | 0.348 | 0.000 |  |  |  |  |  |  |  |  |  |  |
| **5** | Lafitte-sur-Lot, France and Vacaria, Brazil | 0.152 | 0.221 | 0.194 | 0.122 | 0.000 |  |  |  |  |  |  |  |  |  |
| **6** | Campos de Holambra, Brazil | 0.279 | 0.349 | 0.441 | 0.324 | 0.226 | 0.000 |  |  |  |  |  |  |  |  |
| **7** | Argentina and Chile | 0.118 | 0.165 | 0.226 | 0.367 | 0.256 | 0.397 | 0.000 |  |  |  |  |  |  |  |
| **8** | Feicheng and Taigu, China | 0.095 | 0.158 | 0.143 | 0.299 | 0.183 | 0.354 | 0.174 | 0.000 |  |  |  |  |  |  |
| **9** | Yangling, China | 0.154 | 0.228 | 0.253 | 0.431 | 0.299 | 0.508 | 0.209 | 0.073 | 0.000 |  |  |  |  |  |
| **10** | Pulandian, China | 0.109 | 0.177 | 0.112 | 0.333 | 0.210 | 0.428 | 0.205 | 0.073 | 0.144 | 0.000 |  |  |  |  |
| **11** | South Korea | 0.165 | 0.193 | 0.178 | 0.365 | 0.237 | 0.446 | 0.262 | 0.092 | 0.192 | 0.084 | 0.000 |  |  |  |
| **12** | Japan | 0.215 | 0.222 | 0.231 | 0.419 | 0.282 | 0.496 | 0.295 | 0.133 | 0.226 | 0.119 | 0.072 | 0.000 |  |  |
| **13** | South Africa | 0.238 | 0.353 | 0.386 | 0.607 | 0.388 | 0.629 | 0.350 | 0.306 | 0.459 | 0.359 | 0.380 | 0.440 | 0.000 |  |
| **14** | Australia | 0.155 | 0.187 | 0.215 | 0.349 | 0.244 | 0.400 | 0.208 | 0.111 | 0.200 | 0.127 | 0.136 | 0.187 | 0.358 | 0.000 |

**Appendix 2**. Pairwise *F_ST_* values from 14 clusters derived from analysis in Geneland. *F_ST_* values were calculated in GenAlEx.
